# MYB Confers Sorafenib Resistance in Human Leukemia Cells via Inhibiting Ferroptosis Through FTH1 Upregulation

**DOI:** 10.3390/genes16070737

**Published:** 2025-06-26

**Authors:** Xiaoxiao Tao, Yucheng Wang, Siyu Shen, Huiying Fang, Hongkuan Song, Junfang Zhang, Bingshe Han

**Affiliations:** 1 Key Laboratory of Exploration and Utilization of Aquatic Genetic Resources, Ministry of Education, Shanghai Ocean University, Shanghai 201306, China; xiaoxiaotao6222@163.com (X.T.); wanyuch123@163.com (Y.W.); siyushen123@163.com (S.S.); fanghuiying197@163.com (H.F.); m13790874088@163.com (H.S.); jfzhang@shou.edu.cn (J.Z.); 2 National Demonstration Center for Experimental Fisheries Science Education, Shanghai Ocean University, Shanghai 201306, China; 3 Marine Biomedical Science and Technology Innovation Platform of Lin-Gang Special Area, Shanghai 201306, China

**Keywords:** *MYB*, leukemia, sorafenib resistance, *FTH1*, ferroptosis

## Abstract

Background: MYB is a key transcription factor that plays an essential role in regulating hematopoiesis, particularly influencing cell proliferation, differentiation, and apoptosis. It has been extensively implicated in the pathogenesis and progression of leukemia, as well as in determining patient prognosis and responsiveness to chemotherapy. Despite these well-documented roles, the precise molecular mechanisms by which MYB contributes to chemotherapy resistance in leukemia remain largely undefined. Methods: In this study, we investigated the potential role of MYB in regulating ferroptosis, a form of regulated cell death driven by iron-dependent lipid peroxidation, which has recently emerged as a novel therapeutic target in cancer. We overexpressed and knockdown MYB in human leukemia K562 cells and evaluated changes in ferroptosis-related markers, as well as cell proliferation and migration capacities, in the context of treatment with the chemotherapeutic agent sorafenib. Results: Our findings demonstrated that *MYB* overexpression significantly enhanced the resistance of human leukemia cells to sorafenib, while *MYB* knockdown increased their drug sensitivity. Mechanistically, *MYB* was found to upregulate ferritin heavy chain 1 (FTH1), thereby suppressing sorafenib-induced ferroptosis and cell death. Further, *FTH1* knockdown significantly reduced the proliferation and migration ability of K562 cells and enhanced sorafenib-induced ferroptosis. Rescue experiments confirmed that *FTH1* is required for *MYB* induced sorafenib resistance and ferroptosis inhibition in human leukemia cells. Conclusions: Collectively, this study identifies the *MYB-FTH1* axis as a novel regulatory pathway modulating ferroptosis and chemoresistance in leukemia cells, providing potential therapeutic targets for improving treatment precision and preventing disease relapse.

## 1. Introduction

The transcription factor MYB is a key regulator for the hematopoietic process [1], and precise regulation of MYB is necessary for every stage of the development of thymocytes [2], red blood cells [3], B lymphocytes [4], and the self-renewal of hematopoietic stem cells (HSCs) [5]. Aberrant MYB expression plays a critical role in the onset and progression of various hematologic malignancies, particularly leukemia [6]. In addition, MYB dysregulation contributes to drug resistance in leukemia as well as gastrointestinal and breast cancers [7]. Thus, understanding the underlying mechanisms of MYB-mediated drug resistance is of great significance for the treatment of leukemia.

Sorafenib is an orally administered multikinase inhibitor that targets both Raf serine/threonine kinases and various receptor tyrosine kinases [8]. Sorafenib has been approved for liver cancer and kidney cancer [9], and has also shown therapeutic efficacy in acute myeloid leukemia (AML) patients with FLT3-ITD mutations, leading to improved patient survival [10]. In addition, a persistently low percentage of blasts, along with CD3+ cell infiltration in the skin, increased CD8+ lymphocytes in the bone marrow, and elevated expression of COL4A3, TLR9, FGF1, and IL-12 genes have been observed in sorafenib-treated AML patients [11]. A combination of sorafenib and decitabine has been used in preclinical and clinical trials to treat FLT3/ITD-mutated AML [12]. Unfortunately, most patients failed to experience a long-term benefit, largely because of the early occurrence of sorafenib resistance [13]. Since sorafenib can exhibit its anticancer effect through inducing apoptosis and ferroptosis, desensitivity of cancer cells to ferroptosis will lead to resistance.

Ferroptosis is an iron-driven cell death modality characterized by iron accumulation and excessive lipid peroxidation [14]. Ferroptosis dysfunction is involved in diseases such as degenerative diseases [15], solid tumors [16], and leukemia [17]. Ferroptosis is also associated with drug resistance in cancer therapy, and inducing ferroptosis has been demonstrated to reverse drug resistance [18]. Ferritin heavy chain 1 (FTH1) is an iron storage protein with ferroxidase activity, which converts the ferrous form (Fe^2+^) to the ferric form (Fe^3+^) [19]. High expression of *FTH1* can promote tumor cell proliferation and migration and inhibit ferroptosis in leukemia [20]. *FTH1* has also been identified as a key gene associated with poor prognosis in AML [21]. In addition, chemotherapy resistance progression of various diseases is closely related to *FTH1* [22].

In this study, we found that MYB can inhibit sorafenib induced ferroptosis in human leukemia K562 cells through upregulating *FTH1*, conferring sorafenib resistance to leukemia cells. Our results help understanding the mechanism of MYB-mediated resistance to anticancer drugs, and suggest that targeting ferroptosis may be a promising therapeutic strategy for MYB-mediated drug resistance in leukemia.

## 2. Materials and Methods

### 2.1. Cell Culture

The K562, HL-60, and HEK 293T cell lines were obtained from ATCC (Manassas, VA, USA). K562 (CCL-243) and HL-60 (CCL-240) cells were grown in RPMI-1640 Medium (C11875500BT, Gibco, Waltham, MA, USA), while HEK 293T cells (CRL-3216) were maintained in DMEM (C11965500BT, Gibco, USA). All cell lines were supplemented with 10% fetal bovine serum (FBS, 10099141, Gibco, USA) and incubated in a humidified atmosphere at 37 °C with 5% CO_2_.

### 2.2. Plasmid Construction

The pLVX-IRES-neo plasmid (632181, Clontech, Mountain View, CA, USA), containing the sequence of *MYB*, was constructed and used to upregulate *MYB* expression. Two specific short hairpin RNAs (shRNAs) targeting the covalent closed junction of *MYB* and *FTH1* were cloned into the pLKO.1-puro plasmid (8453, Addgene, Watertown, MA, USA). Sequences of the above oligos are listed in Table 1.

### 2.3. RNA Extraction and Quantitative Real-Time PCR (RT-qPCR)

RNA was extracted using TRIzol (15596018, Invitrogen, Carlsbad, CA, USA). The RNA was converted into cDNA using the PrimeScript™ RT Reagent Kit (RR037A, Takara, Shiga, Japan) according to the manufacturer’s instructions. RT-qPCR was performed in a LightCycler 480 II instrument using SYBR Green Master Mix (AQ601, TransGen Biotech, Beijing, China). *GAPDH* was used as the internal control, and the relative expression levels of specific genes were calculated using the 2^−ΔΔCt^ method. The primers used for *RT-qPCR* are listed in Table 2.

### 2.4. Lentivirus Preparation and Infection

Lentiviruses for *MYB* overexpression and *MYB* or *FTH1* knockdown were produced by co-transfecting constructed plasmids and the packaging plasmids pCMV-dR8.91 and pCMV-VSV-G into HEK 293T cells using Lipofectamine 3000 Reagent (L3000015, Thermo Scientific, Waltham, MA, USA) for 72 h. Culture supernatants containing lentivirus were collected, filtered, and concentrated. K562 cells were infected with lentivirus in the presence of 8 µg/mL polybrene (Sigma-Aldrich, St. Louis, MO, USA).

### 2.5. Western Blot

Cells were lysed using RIPA buffer (P0013B, Beyotime, Shanghai, China), and lysates were centrifuged at 12,000× *g* for 10 min at 4 °C to collect the supernatant. Equal amounts of protein were separated by SDS-PAGE, and transferred to 0.2 μm PVDF membranes (LC2002, Thermo Scientific, USA). Membranes were blocked with 5% (*w*/*v*) skim milk at room temperature for 2 h and then incubated overnight at 4 °C with primary antibodies. After washing, membranes were incubated with HRP-conjugated secondary antibodies at room temperature for 2 h. Protein bands were visualized using Clarity™ Western ECL Substrate (170–5061, Bio-Rad, Hercules, CA, USA). Primary antibodies used included anti-c-MYB (sc-74512, Santa Cruz Biotechnology, Dallas, TX, USA), anti-FTH1 (MA5-43829, Thermo Fisher Scientific, USA), and anti-beta actin (A13685, HUABIO, Hangzhou, China); the secondary antibody was HRP-conjugated goat anti-mouse IgG (31420, Thermo Scientific, USA).

### 2.6. Determination of Reactive Oxygen Species (ROS)

K562 cells were collected by centrifuge and ROS levels were determined using a Reactive Oxygen Species Assay Kit (S0033S, Beyotime, China). The DCFH-DA was diluted with serum-free medium to reach a final concentration of 10 μM. Then, 1 mL of DCFH-DA was added into cells for incubation at 37 °C for 30 min, blending every 3 min. Cells were washed (×3 times) and the absorbance was determined at an excitation wavelength of 485 nm and an emission wavelength of 528 nm using a microplate reader (SYNERGY2, BIO-Tek, Shoreline, WA, USA).

### 2.7. Determination of Fe^2+^

K562 cells were washed three times with cell staining buffer. Fe^2+^ levels were determined using a Cell Ferrous Iron (Fe^2+^) Fluorometric Assay Kit (MA0647, MeilunBio, Dalian, China). The ferrous ion probe was diluted with cell staining buffer to reach a final concentration of 2 μM. Then, 1 mL of staining working solution was added into cells for incubation at 37 °C for 45 min. Absorbance was determined at an excitation wavelength of 543 nm and an emission wavelength of 580 nm using a microplate reader (SYNERGY2, BIO-Tek, USA).

### 2.8. CCK8 Assay

Cell proliferation was assessed using the Enhanced Cell Counting Kit-8 (C0042, Beyotime, China). Briefly, cells were plated in 96-well plates and incubated for 24, 48, 72, and 96 h. Then, 10 µL of CCK8 solution was added, and the cells were incubated at 37 °C for 2 h. Absorbance was measured at 450 nm using a microplate reader (ReadMax1900, Shanpu, Wuxi, China).

### 2.9. Transwell Assay

K562 cells (5 × 10^5^/mL) were seeded into the upper chamber of 24-well Transwell plates with 8 μm pore size (CLS3422, Corning, Corning, NY, USA), while the lower chamber was filled with RPMI-1640 medium containing 10% FBS as a chemoattractant. After incubating for 16 h, non-migrating cells on the upper side of the chamber were removed by scrubbing, and migrating cells on the lower side were fixed with 4% paraformaldehyde and stained with Giemsa (E607315-0001, Sangon Biotech, Shanghai, China). The number of migrating cells was assessed in 3 random microscope fields.

### 2.10. Statistical Analysis

Each experiment was performed thrice for technical consistency. Statistical analyses were performed using GraphPad Prism version 8.0. Student’s *t*-test was used to determine the significance of differences between two groups.

## 3. Results

### 3.1. MYB Induces Drug Resistance in Human Leukemia Cells

**Figure 1:**
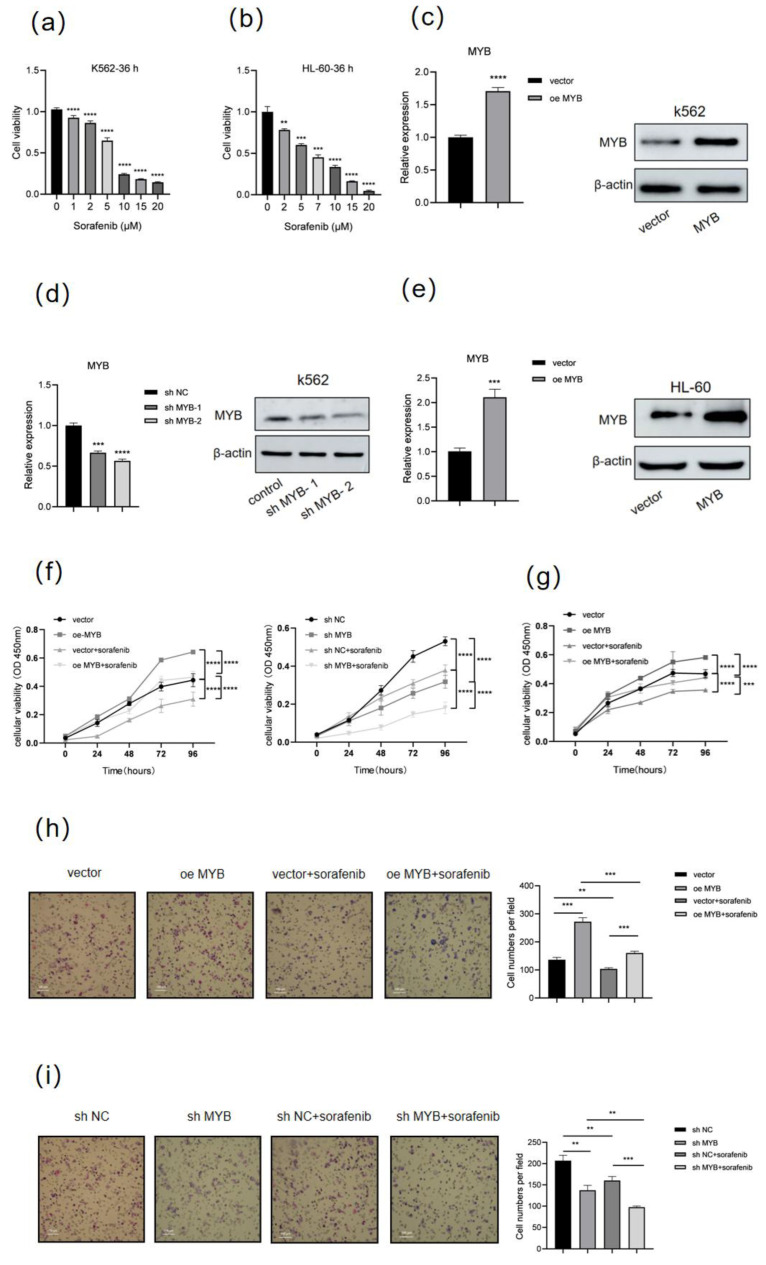
MYB induces sorafenib resistance in human leukemia cells. (**a**) K562 cells were treated with sorafenib at the indicated doses (0, 1, 2, 5, 10, 15, 20 μM) for 36 h, and cell viability was measured by a CCK8 assay. (**b**) HL-60 cells were treated with sorafenib at indicated doses for 36 h, and cell viability was measured by a CCK8 assay. (**c**,**d**) K562 cells were infected with control lentivirus (vector), lentivirus expressing MYB (oe MYB), negative control shRNA (sh NC), or MYB-targeting shRNA (sh MYB-1 and sh MYB-2) for 48 h, then the expression level of MYB was determined by RT-qPCR and Western blot. (**e**) HL-60 cells were infected with lentivirus expressing MYB or control lentivirus (vector) for 48 h, then MYB expression was evaluated by RT-qPCR and Western blot. (**f**) K562 cells were treated with 5 μM sorafenib for 36 h after infection with the indicated lentivirus, and cell viability was measured by a CCK8 assay. (**g**) HL-60 cells were treated with 5 μM sorafenib for 36 h after MYB overexpression, and cell viability was measured by a CCK8 assay. (**h**,**i**) A Transwell migration assay was performed on K562 cells after MYB overexpression or knockdown and sorafenib treatment. Scale bar: 100 μm. Data for graphing were obtained from the means ± SD of three independent experiments (*n* = 3). *p* values were calculated using Student’s *t*-test (** *p* < 0.01, *** *p* < 0.001, **** *p* < 0.0001).

To determine the sensitivity of human leukemia cells to sorafenib treatment, K562 and HL-60 cells were treated with different concentrations of sorafenib for 36 h, then cell viability was detected using a CCK8 assay (Figure 1a,b). In line with expectations, sorafenib treatment led to a dose-dependent decrease in the viability of K562 and HL-60 cells. Then, we selected 5 μM sorafenib for further study. To explore the effect of *MYB* on sorafenib treatment in K562 and HL-60 cells, MYB was either overexpressed or silenced using two different shRNAs (shMYB-1 and shMYB-2) (Figure 1c–e). The sequences of these shRNAs are listed in Table 1. shMYB-2 (hereafter referred to as shMYB) was used for further experiments. The CCK8 assay showed *MYB* overexpression partially reversed the decrease in cell viability caused by sorafenib, while *MYB* knockdown enhanced the inhibitory effect of sorafenib in K562 cells (Figure 1f). Similarly, *MYB* overexpression attenuated the sorafenib-induced reduction in cell viability in HL-60 cells. (Figure 1g). The Transwell assay showed that sorafenib inhibited the migration of K562 cells, *MYB* overexpression partially reversed the inhibition of cell migration caused by sorafenib, and *MYB* knockdown enhanced the inhibitory effect of sorafenib on the migration (Figure 1h,i). The above results show that *MYB* confers sorafenib resistance in human leukemia cells.

### 3.2. MYB Inhibits Ferroptosis in K562 Cells

**Figure 2:**
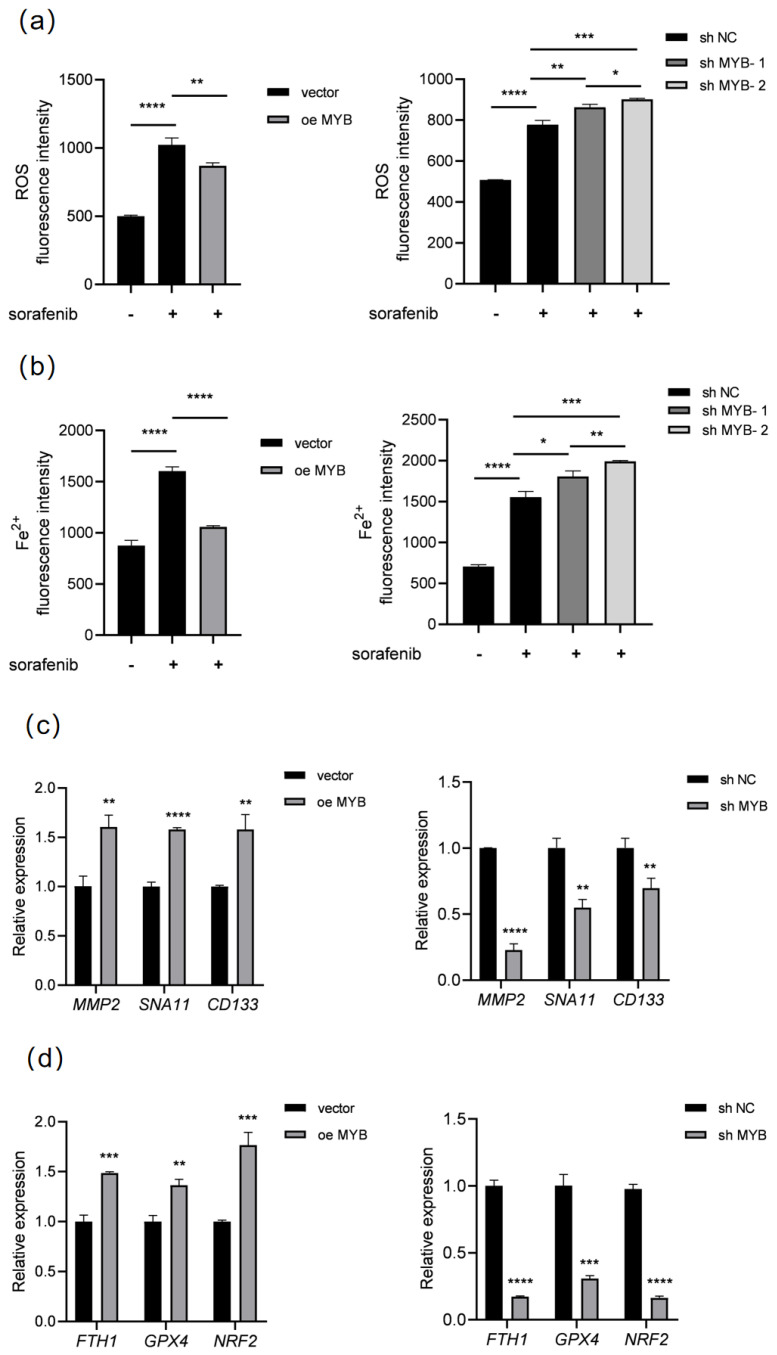
MYB inhibits ferroptosis in K562 cells. K562 cells were treated with 5 μM sorafenib for 36 h after infection with a control lentivirus (vector), lentivirus expressing MYB, negative control shRNA (sh NC), or MYB targeting shRNA (sh MYB-1 and sh MYB-2), then ROS (**a**) and Fe^2+^ levels (**b**) were determined. K562 cells were infected with the indicated lentivirus for 48 h, then the expression of proliferation and migration related genes (**c**) and ferroptosis related genes (**d**) was examined using RT-qPCR. Data for graphing were obtained from the means ± SD of three individual experiments (*n* = 3), and *p* values were calculated using Student’s *t*-test (* *p* < 0.05, ** *p* < 0.01, *** *p* < 0.001, **** *p* < 0.0001).

To investigate the underlying mechanism, we performed RNA-seq in K562 cells after *MYB* overexpression; the results showed that MYB is closely related to the regulation of ferroptosis, and sorafenib is also a ferroptosis inducer. To further confirm the role of MYB in ferroptosis, we detected ROS and Fe^2+^ in K562 cells after *MYB* overexpression and knockdown. As shown in Figure 2a,b, sorafenib treatment increased the levels of ROS and Fe^2+^, while *MYB* overexpression restrained the increase of ROS and Fe^2+^ induced by sorafenib in K562 cells. In contrast, sh MYB led to a further increase in the levels of ROS and Fe^2+^ in sorafenib-treated K562 cells. This indicates that MYB inhibits sorafenib induced ferroptosis in K562 cells.

Meanwhile, the expression of proliferation and migration-related genes were detected. As shown in Table 2, RT-qPCR showed that *MYB* overexpression significantly upregulated *MMP2*, *SNA11*, and *CD133*, while sh MYB downregulated these genes (Figure 2c). Subsequently, we further analyzed the expression of ferroptosis-related genes. RT-qPCR showed that *MYB* overexpression significantly upregulated *FTH1*, *GPX4*, and *NRF2*, three negative regulators of ferroptosis, when sh MYB significantly downregulated these genes (Figure 2d). Since FTH1 is an important negative regulator of ferroptosis, the above data show that MYB can inhibit ferroptosis through regulating ferroptosis-related genes and confer resistance to sorafenib in K562 cells.

### 3.3. FTH1 Knockdown Induces Ferroptosis and Enhances Sorafenib Sensitivity in K562 Cells

**Figure 3:**
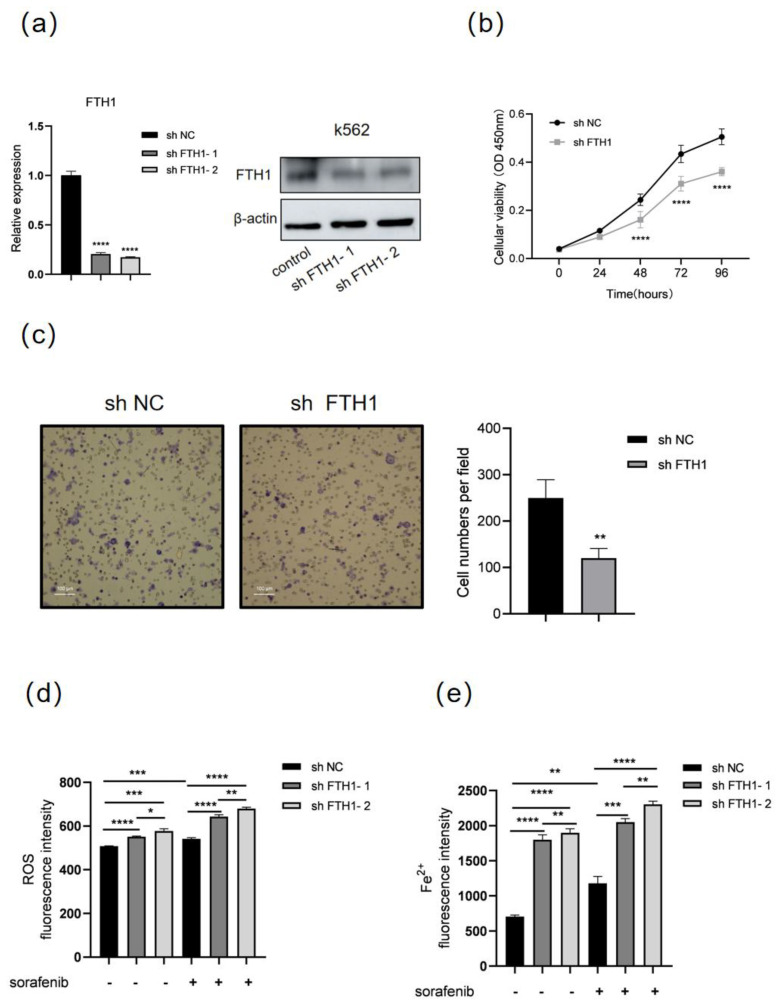
*FTH1* knockdown induces ferroptosis and enhances sorafenib sensitivity in K562 cells. K562 cells were infected with lentivirus expressing negative control shRNA (sh NC) or FTH1-targeting shRNA (sh FTH1-1 and sh FTH1-2) for 48 h, then RT-qPCR, Western blot (**a**), CCK8 assay (**b**), and Transwell assay (**c**) were performed. Scale bar: 100 μm. K562 cells were treated with 5 μM sorafenib for 36 h after infected with the indicated sh RNA lentivirus (sh FTH1-1 and sh FTH1-2), then ROS levels (**d**) and Fe^2+^ levels (**e**) were measured. Data for graphing were obtained from the means ± SD of three individual experiments (*n* = 3), and *p* values were calculated using Student’s *t*-test (* *p* < 0.05, ** *p* < 0.01, *** *p* < 0.001, **** *p* < 0.0001).

Next, we investigated the role of FTH1 in the proliferation and migration of K562 cells. *FTH1* was knocked down by a shRNA (sh FTH1-1 and sh FTH1-2)-expressing lentivirus in K562 cells. RT-qPCR and Western blot showed that *FTH1* was successfully knocked down (Figure 3a). shFTH1-2 (hereafter referred to as sh FTH1) was selected for subsequent experiments. CCK8 assays and Transwell assays showed that knocking down *FTH1* significantly inhibited the proliferation and migration of K562 cells (Figure 3b,c), suggesting that FTH1 plays a vital role in the growth and migration of K562 cells. Next, we explored the role of FTH1 in ferroptosis and sorafenib sensitivity of K562 cells. The results showed that ROS increased significantly after *FTH1* knockdown or sorafenib treatment, and *FTH1* knockdown further enhanced the elevation of ROS and Fe^2+^ caused by sorafenib treatment (Figure 3d,e). These data indicate that FTH1 can support the proliferation and migration of K562 cells, and inhibition of FTH1 causes decreased proliferation and migration and enhanced sorafenib sensitivity in K562 cells.

### 3.4. MYB Inhibits Ferroptosis and Induces Sorafenib Resistance Through FTH1 in Leukemia Cells

**Figure 4:**
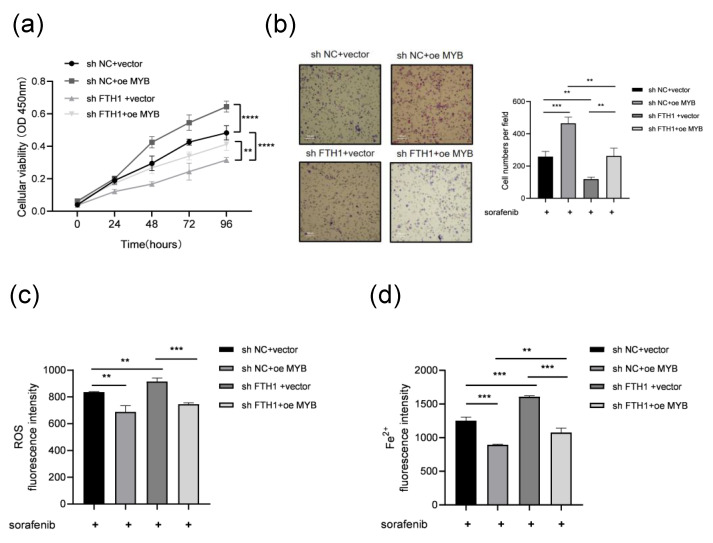
MYB inhibits ferroptosis and induces sorafenib resistance through FTH1 in K562 cells. K562 cells coinfected with the indicated lentivirus were treated with 5 μM sorafenib for 36 h, then a CCK8 assay (**a**) and Transwell assay (**b**) were performed, and ROS levels (**c**) and Fe^2+^ levels (**d**) were examined. Scale bar: 100 μm. Data for graphing were obtained from the means ± SD of three individual experiments (*n* = 3), and *p* values were calculated using Student’s *t*-test (** *p* < 0.01, *** *p* < 0.001, **** *p* < 0.0001).

To investigate the role of FTH1 in MYB-induced sorafenib resistance in leukemia cells, *FTH1* was knocked down in K562 cells with *MYB* overexpression prior to sorafenib treatment. CCK8 and Transwell assays demonstrated that *MYB* overexpression significantly promoted the proliferation and migration of K562 cells, whereas *FTH1* knockdown markedly suppressed these effects. When *FTH1* knockdown and *MYB* overexpression occurred simultaneously, *FTH1* knockdown could partially abolished the increase in cell proliferation and migration caused by *MYB* overexpression (Figure 4a,b). This finding could indicate that *MYB* promotes the proliferation and migration of K562 cells via *FTH1*. Meanwhile, we detected ROS and Fe^2+^ in K562 cells after *FTH1* knockdown and *MYB* overexpression. The results show that *MYB* overexpression reduced the ROS level and Fe^2+^ content of K562 cells, while *FTH1* knockdown increased the ROS level and Fe^2+^ content of K562 cells. Meanwhile, *FTH1* knockdown abolished the reduction of cellular ROS levels and Fe^2+^ content caused by *MYB* overexpression (Figure 4c,d). The above data indicate the presence of MYB-induced sorafenib resistance through the upregulation of FTH1 and inhibition of ferroptosis.

## 4. Discussion

MYB functions as a pivotal regulator of hematopoiesis by modulating various cellular processes such as proliferation [23], differentiation, and programmed cell death [5]. Abnormal expression of MYB is closely related to leukemia progression, poor prognosis, and anticancer drug resistance [7]. It has been reported that MYB silencing reduces the resistance of AML cells to doxorubicin [24]. Our work here shows that MYB can confer resistance to sorafenib via upregulating FTH1 and inhibiting sorafenib-induced ferroptosis in human leukemia. The above results support that targeting the ferroptosis pathway can overcome MYB-mediated drug resistance in leukemia.

It is well known that MYB is highly expressed in various leukemia cells, which often leads to drug resistance. Knockdown of MYB sensitized acute lymphoblastic leukemia (ALL) cells to doxorubicin and 6-mercaptopurine by downregulating anti-apoptotic BCL2 [25]. In ovarian cancer (OC) cells, MYB overexpression induces cisplatin resistance by activating the NF-κB and STAT3 signaling pathways, whereas MYB silencing or inhibition restores cisplatin sensitivity [26]. Here, we showed that MYB inhibits sorafenib-induced ferroptosis in human leukemia, desensitizing these cells to sorafenib. The above results demonstrate that MYB can induce drug resistance to multiple drugs via multiple pathways, and targeting these pathways will help overcoming MYB-related drug resistance in leukemia.

Ferroptosis is frequently dysregulated in cancers and closely related to drug resistance. Ferroptosis induction can alleviate sorafenib resistance in various cancers [27]. FTH1 prevents iron-dependent lipid peroxidation by storing free iron and attenuating ferroptosis [28]. FTH1 promotes leukemia cell proliferation via the ferroptosis pathway, serving as a potential risk factor influencing the prognosis of pediatric non-M3 AML [21]. High FTH1 expression is usually accompanied by the activation of NF-κB-related genes, thereby reducing the sensitivity of AML cells to chemotherapy [29]. This is consistent with our findings that *FTH1* knockout inhibits cell proliferation and migration while enhancing the effect of sorafenib, thereby inducing ferroptosis in leukemia cells.

In tumor progression, multiple key genes are involved in cell proliferation and migration. Matrix metalloproteinase-2 (*MMP-2*) enhances invasion and metastasis in cancer cells by degrading the extracellular matrix (ECM) and promoting angiogenesis [30]. Zinc finger transcription factor (*SNA11*) induces epithelial–mesenchymal transition (EMT) in various cancers and promotes cell migration and invasion [31]. Cell membrane glycoprotein (*CD133*), is closely related to cancer cell proliferation, autophagy, and apoptosis [32]. Glutathione peroxidase 4 (*GPX4*) can effectively remove lipid peroxides and inhibit ferroptosis [33]. Nuclear factor erythroid 2-related factor 2 (*NRF2*), as a core regulator of cellular antioxidant stress, can activate multiple anti-ferroptosis pathways [34]. This study found that MYB can significantly upregulate proliferation and migration-related genes (*MMP2*, *SNA11*, *CD133*) and negative regulatory genes of ferroptosis (*FTH1*, *GPX4*, *NRF2*). These data indicate that MYB supports cell survival through multiple downstream target genes.

The regulation of FTH1 is important for ferroptosis. METTL1 can upregulate mature miR-26a-5p, which then inhibits *FTH1* mRNA translation efficiency; the decrease of FTH1 in turn increases ferroptosis and promotes chemotherapy sensitivity in osteosarcoma cells [35]. LncRNA CACNA1G-AS1 can upregulate *FTH1* to inhibit ferroptosis and promote malignant phenotypes in ovarian cancer cells [36]. The circPIAS1/NUPR1 axis suppresses ferroptosis activity in hepatocellular carcinoma (HCC) cells by upregulating *FTH1*, thereby accelerating HCC progression [37]. Here, we showed that *MYB* can upregulate *FTH1*, inhibit ferroptosis in human leukemia cells. Consistent with our findings, previous CUT&RUN data indicate that MYB binds directly to the promoter region of the FTH1 gene in K562 cells, suggesting that MYB directly regulates FTH1 transcription [38]. Collectively, these findings show that FTH1 is under the control of multiple upstream regulators.

Since FTH1 and MYB inhibition can sensitize human leukemia cells to sorafenib in vitro, combination therapy of sorafenib with FTH1 or MYB inhibitor may overcome drug resistance in sorafenib-resistant AML patients; further research using primary AML patient samples and in vivo models is required to support combining FTH1 or MYB inhibitors with sorafenib as a therapeutic strategy in the treatment of AML.

In summary, MYB plays a critical role in regulating chemoresistance through multiple mechanisms in various cancers, particularly in leukemia. Our results showed that *MYB* inhibits sorafenib-induced ferroptosis by positively regulating *FTH1* expression, thereby enhancing the resistance of K562 cells to sorafenib. These findings emphasize the key role of *MYB*-*FTH1* in inhibiting chemotherapy-induced ferroptosis and drug resistance.

## Figures and Tables

**Table 1:** The sequences of shRNAs.

Name	Forward Primer (5′ → 3′)	Reverse Primer (5′ → 3′)	TRCN Number
sh MYB-1	CCGGAACAGAATGGAACAGATGACCTCGAGGTCATCTGTTCCATTCTGTT TTTTTG	AATTCAAAAAAACAGAATGGAACAGATGACCTCGAGGTCATCTGTTCCATTCTGTT	TRCN0000009853
sh MYB-2	CCGGGCTCCTAATGTCAACCGAGAACTCGAGTTCTCGGTTGACATTAGGAGCTTTTTG	AATTCAAAAAGCTCCTAATGTCAACCGAGAACTCGAGTTCTCGGTTGACATTAGGAGC	TRCN0000288659
sh FTH1-1	CCGGGCCGAATCTTCCTTCAGGATACTCGAGTATCCTGAAGGAAGATTCGGCTTTTTG	AATTCAAAAAGCCGAATCTTCCTTCAGGATACTCGAGTATCCTGAAGGAAGATTCGGC	TRCN0000029433
sh FTH1-2	CCGGCCTGTCCATGTCTTACTACTTCTCGAGAAGTAGTAAGACATGGACAGGTTTTTG	AATTCAAAAACCTGTCCATGTCTTACTACTTCTCGAGAAGTAGTAAGACATGGACAGG	TRCN0000029432
sh NC	CCGGGCAAGCTGACCCTGAAGTTCATCTCGAGATGAACTTCAGGGTCAGCTTGCTTTTTG	AATTCAAAAAGCAAGCTGACCCTGAAGTTCATCTCGAGATGAACTTCAGGGTCAGCTTGC	

**Table 2:** Primer sequences for quantitative real-time PCR.

Name	Forward Primer (5′ → 3′)	Reverse Primer (5′ → 3′)
MYB	ATACATGAACGAGGAGCAGCG	TCATGGTCTGACTGTGGGAT
FTH1	TGAAGCTGCAGAACCAACGAGG	GCACACTCCATTGCATTCAGCC
GPX4	ACAAGAACGGCTGCGTGGTGAA	GCCACACACTTGTGGAGCTAGA
NRF2	GCTCAAACTTAGGGGCTCCG	GAAGTTGCGGGAAGGTCTGG
MMP2	ACCTGGATGCCGTCGGGAC	TGTGGCAGCACCAGGGCAGC
SNA11	TCGGAAGCCTAACTACAGCGA	AGATGAGCATTGGCAGCGAG
CD133	GCCACCGCTCTAGATACTGC	TGTTGTGATGGGCTTGTCAT
GAPDH	GGAAGGTGAAGGTCGGAGTCA	GTCATTGATGGCAACAATATCCACT

## Data Availability

All the data generated or analyzed during this study are included in the published article.
